# Memory B Cell Antibodies to HIV-1 gp140 Cloned from Individuals Infected with Clade A and B Viruses

**DOI:** 10.1371/journal.pone.0024078

**Published:** 2011-09-08

**Authors:** Hugo Mouquet, Florian Klein, Johannes F. Scheid, Malte Warncke, John Pietzsch, Thiago Y. K. Oliveira, Klara Velinzon, Michael S. Seaman, Michel C. Nussenzweig

**Affiliations:** 1 Laboratory of Molecular Immunology, The Rockefeller University, New York, New York, United States of America; 2 Charite Universitaetsmedizin, Berlin, Germany; 3 Universitätsklinikum Hamburg-Eppendorf, Hamburg, Germany; 4 Department of Biology, Chemistry, Pharmacy, Freie Universität Berlin, Berlin, Germany; 5 Medical School of Ribeirao Preto/USP, Department of Genetics, National Institute of Science and Technology for Stem Cells and Cell Therapy and Center for Cell-Based Therapy, Ribeirao Preto, Brazil; 6 Beth Israel Deaconess Medical Center, Boston, Maryland, United States of America; 7 Howard Hughes Medical Institute, The Rockefeller University, New York City, New York, United States of America; University of Pennsylvania, United States of America

## Abstract

Understanding the antibody response to HIV-1 in humans that show broad neutralizing serologic activity is a crucial step in trying to reproduce such responses by vaccination. Investigating antibodies with cross clade reactivity is particularly important as these antibodies may target conserved epitopes on the HIV envelope gp160 protein. To this end we have used a clade B YU-2 gp140 trimeric antigen and single-cell antibody cloning methods to obtain 189 new anti-gp140 antibodies representing 51 independent B cell clones from the IgG memory B cells of 3 patients infected with HIV-1 clade A or B viruses and exhibiting broad neutralizing serologic activity. Our results support previous findings showing a diverse antibody response to HIV gp140 envelope protein, characterized by differentially expanded B-cell clones producing highly hypermutated antibodies with heterogenous gp140-specificity and neutralizing activity. In addition to their high-affinity binding to the HIV spike, the vast majority of the new anti-gp140 antibodies are also polyreactive. Although none of the new antibodies are as broad or potent as VRC01 or PG9, two clonally-related antibodies isolated from a clade A HIV-1 infected donor, directed against the gp120 variable loop 3, rank in the top 5% of the neutralizers identified in our large collection of 185 unique gp140-specific antibodies in terms of breadth and potency.

## Introduction

A significant fraction of the patients infected with HIV-1 develop broadly neutralizing serologic activity 2–3 years after infection [1], [2], [3], [4], [5], [6], [7], [8]. Although these antibodies do not protect infected patients, they put selection pressure on the virus [9]. Additionally, and more importantly, passive transfer of broadly neutralizing antibodies to monkeys effectively protects them against SHIV infection [10], [11], [12], [13], [14], [15], [16], [17], [18], [19]. Therefore, it has been proposed that vaccines that elicit such antibodies may be protective against infection in humans [20], [21], [22], [23], [24].

Despite a wealth of serologic information and significant efforts to obtain representative broadly neutralizing antibodies, there have been few systematic molecular studies of the anti-HIV-1 antibody response [25], [26]. Nevertheless, several broadly neutralizing antibodies (bNAbs) to HIV-1 gp140 have been isolated including a group that binds to gp120 (b12, 2G12, PG9/PG16, HJ16 and VRC01) [26], [27], [28], [29], [30] and a group that is specific for gp41 (2F5, 4E10 and Z13) [31], [32], [33]. The precise way in which these unique and potentially important antibodies relate to the serologic responses remains unclear. The serologic response to HIV-1 is polyclonal and targets both internal and viral surface proteins, but only antibodies directed against the HIV envelope spike, gp160, mediate viral neutralization [24].

In order to examine the memory B cell compartment of HIV-1 infected patients we developed a method to directly clone antibodies from anti-gp140 specific B cells [25], [34], [35]. Initially, six elite controllers and slow progressors infected with HIV-1 clade B were examined [25]. We found that the IgG memory antibody response to the gp140 HIV envelope protein in those patients was composed of differentially expanded clones (22 to 50 *per* patient) that target a number of different gp120- and gp41-epitopes [25], [36], including a new epitope, “CD4bs/DMR” which is closely apposed to the CD4 binding site (CD4bs), conserved between virus variants and required for optimal HIV infectivity [37]. Although no single monoclonal antibody mirrored the broad neutralizing activity in serum, high concentrations of pools of antibodies from 2 of the 4 patients tested reconstituted the initial serologic neutralizing activity [25]. Significantly, in addition to their specific high affinity binding to HIV gp140, 75% of the 134 antibodies were also polyreactive [38]. We have proposed that this property increases relative antibody affinity to the HIV virion by allowing bivalent heteroligation of one high-affinity anti-gp140 combining site and a second low-affinity polyreactive ligand [38].

Here, we extended our study of the human memory B-cell response to HIV by characterizing 189 new anti-gp140 specific antibodies representing 51 independent clones isolated from two HIV-1 clade A and one clade B infected donors with broad neutralizing serologic activity, none of which is an elite controller. The antibody response to gp140 in these patients is highly polyreactive and targets a diverse group of HIV-1 epitopes including “CD4bs/DMR.” Although each individual antibody neutralizes only a limited number of viral strains, many show neutralizing activity to different tier 1 viruses and a limited number of tier 2 viruses.

## Results

### Anti-gp140 antibodies from HIV-1 patients infected with clade A and B viruses

**Figure 1:**
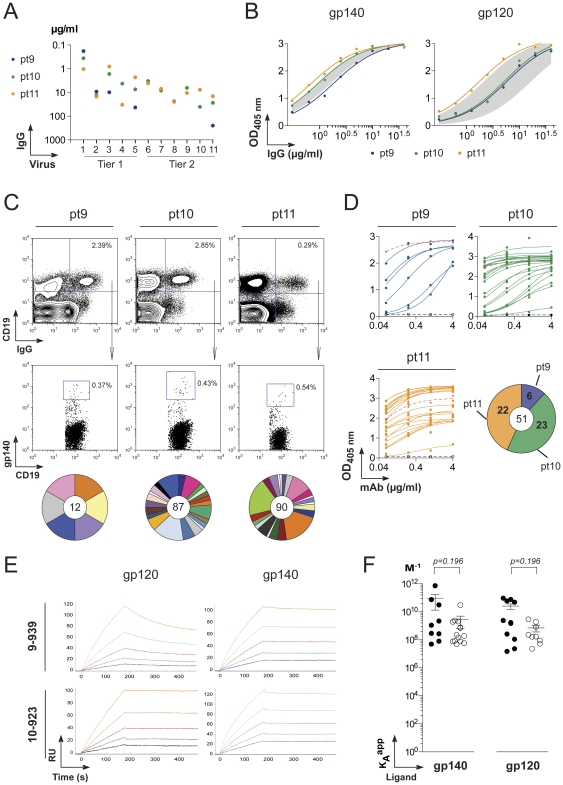
Production of anti-gp140 HIV antibodies from single memory B cells. **A.** Neutralization activity of purified IgGs from HIV-infected patients (pt9-11) sera measured by TZM-bl assay. The *y*-axis shows the IgG concentration required to achieve IC_50_ for each viruses indicated on the *x*-axis: 1, MW965.26, 2, DJ263.8; 3, SF162.LS; 4, SS1196.1; 5, BaL26; 6, 6535,3; 7, RHPA4259.7; 8, SC422661.8; 9, TRO.11; 10, PVO.4 for pt9 and pt10, and CAAN5342.A2 for pt11; 11, YU2.DG. **B.** IgG antibody reactivity of pt9-11 patient sera against YU-2 trimeric gp140 and gp120 proteins determined by ELISA. The grey area indicates the reactivity range of the serum IgGs from previously studied clade B HIV-infected patients (pt1 to pt6) [25] used as comparison. **C.** Flow cytometry plots show the staining of patient PBMC with gp140 protein, anti-CD19 and anti-IgG antibodies. Gp140-rective IgG memory B cells were identified as gp140+IgG+CD19+ cells in the lymphocyte gate. The pie charts below the flow cytometry plots show for each patient the expansions of gp140-specific B-cell clones. The total number of memory B-cell antibodies is indicated in the center, each pie slice represents a clonal family and the area of the slice is proportional to the number of clonal relatives. Each clonal family is represented by the same color and unique antibodies that are not members of a clonal family are not colored. **D.** Gp140-antibody binding by ELISA of anti-gp140 monoclonal antibodies isolated from gp140+IgG+CD19+ cells in HIV-infected patients. The red and black dotted lines show positive (b12) [27] and negative (mGO53) [34] antibody controls. All the experiments were performed at least in duplicate. Representative data are shown. The pie chart indicates the numbers of gp140-reactive antibodies produced from each HIV+ donor with the total number in the center. **E.** Antibody binding to gp140 and gp120 measured by surface _lasmon resonance (SPR). The SPR sensorgrams for antibody binding to gp140 and gp120 overtime are shown for 10-939 and 10-923 as example. The antibodies were tested at concentrations ranging from 2.5 nM (black curve) to 40 nM (orange curve). RU, response units. **F.** Apparent affinities (K_A_^app^) to gp140 and gp120 ligands determined by SPR (as shown in **E**) for the anti-gp140 antibodies isolated from clade A HIV-infected African patients (filled circles) in comparison to the previously studied gp140-reactive antibodies produced from clade B HIV+ donors (open circles) [25]. Student t-test shows no statistical difference.

Three HIV-1 infected donors with heterogenous levels of high serologic neutralizing activity were studied (Figures 1A, Table S1). Two were African donors infected with clade A HIV viruses (pt9 and pt10) and the other, a Caucasian donor, with a clade B virus (pt11). Purified serum IgG from these patients showed similar levels of ELISA binding activity to artificially trimerized YU-2 gp140 (gp140) and YU-2 gp120 as previously studied elite controller HIV patients (Figure 1B) [25]. Consistent with the ELISA results, we found that 0.37–0.54% of the peripheral IgG^+^ B cells from the three patients bound YU-2 gp140 as measured by flow cytometry [35] (Figure 1C). Despite relatively high titers of neutralizing antibodies, one of the patients, pt11, showed a dramatic reduction in the overall frequency of IgG^+^ B cells in a manner consistent with memory compartment exhaustion (Figure 1C) [39].

Immunoglobulin heavy and light chains were cloned from cDNA libraries prepared from single gp140-specific memory B cells isolated by flow cytometry [35], [40]. Of the 189 antibodies obtained, 182 were members of 44 variably expanded clonal families (Figure 1C and Table S2). Since nearly all of the gp140-binding B cells in clade B HIV-infected donors were members of expanded clones of B cells [25], we selected representative members of each clonal family for expression and further analysis (Table S2).

All 51 of the selected antibodies bound to YU-2 gp140 as measured by ELISA (Figure 1D). Moreover, analysis of the antibody affinity by surface plasmon resonance showed that antibodies isolated from clade A HIV patients bound with high affinity to clade B YU-2 gp140 and gp120 ligands (Figures 1E and S1). The apparent affinity constants (K_A_^app^) of the antibodies varied between 4.9×10^−7^ to 7.3×10^−11^ for gp140 and 1.5×10^−7^ to 9.4×10^−10^ gp120 (Figure S1C). Thus the K_A_^app^ of the antibodies from clade A infected patients were comparable to the values obtained from clade B infected individuals [25] (Figure 1F).

### Immunoglobulin gene repertoire

**Figure 2:**
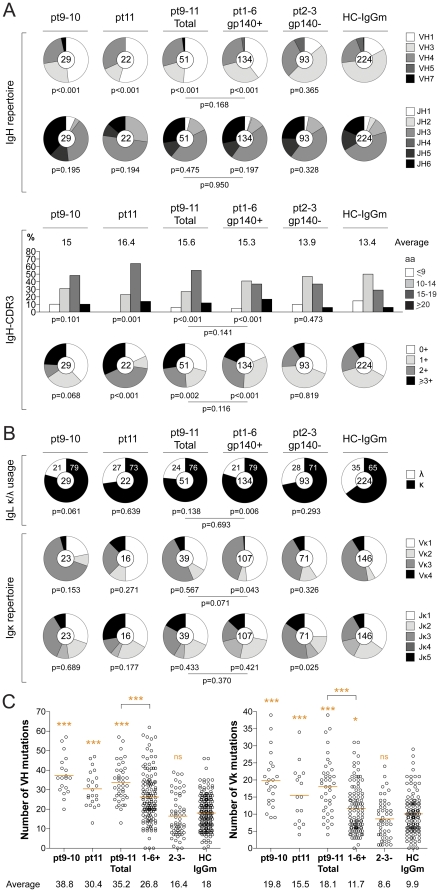
Ig gene repertoire of gp140-specific IgG memory B-cell antibodies. The IgH and IgL chain gene features of anti-gp140 memory B cell antibodies isolated from clade A (pt9 and pt10 shown as a group) and B (pt11) HIV+ patients are shown in comparison to previously published anti-gp140 (pt1-6 gp140+) and non-gp140 reactive (pt2-3 gp140-) antibodies [25] and control antibodies from IgG memory B cells from healthy donors (HC-IgGm) [41]. **A.** IgH V and J gene usages, CDR3 length and CDR3 positive charge numbers from gp140-specific memory B cell antibodies in clade A and B HIV-infected patients compared to controls. The number of antibody sequences analyzed is indicated in the center of each pie chart. The average of IgH CDR3 length is indicated above each histogram. *P*-values indicated below the pie charts or histograms and the lines were calculated by comparison to the HC-IgGm and anti-gp140 (pt1-6 gp140+) control antibodies, respectively [25], [41]. **B.** IgL κ/λ usage, V and J gene usages for Igκ in gp140-specific memory B cell and control antibodies as shown in A. **C.** The numbers of mutations in VH and Vκ genes in gp140-specific memory B cell and control antibodies as shown in **A.** The average number of mutations in VH and Vκ genes is indicated below each dot plot. The *p*-values were determined by comparison to HC-IgGm and anti-gp140 (pt1-6 gp140+) control antibodies [25], [41] using unpaired student's t-test.

Features of the antibodies produced from clade A (pt9 and pt10) and clade B (pt11) infected patients were analyzed individually and as a group (Table S2). Similar to the clade B elite controllers, the antibodies from the new patients were highly biased towards VH1 usage, and enriched for longer, charged IgH complementary-determining region 3 (CDR3) (Figure 2A). In addition, the heavy- and light-chain variable region genes (VH and Vκ, respectively) from anti-gp140-reactive antibodies were highly mutated when compared to non gp140-reactive antibodies isolated from clade B infected elite controllers [25] or historical controls [25], [41]. Indeed, the average number of VH and Vκ mutations in pt9-11 patients was significantly higher than in previously studied pt1-6 patients [25] (35.2 *vs* 26.8 for VH, *p*<0.0001 and 18.1 *vs* 11.7 for Vκ, *p*<0.0001) (Figure 2C). Moreover, as observed for the initial group we found a bias to Igκ light chain usage (Figure 2B). We conclude that the Ig repertoire of anti-gp140 antibodies found in clade A and B HIV-1 infected patients is similar to that described for elite controllers and slow progressors.

### gp140-epitopes targeted by IgG memory antibodies

**Figure 3:**
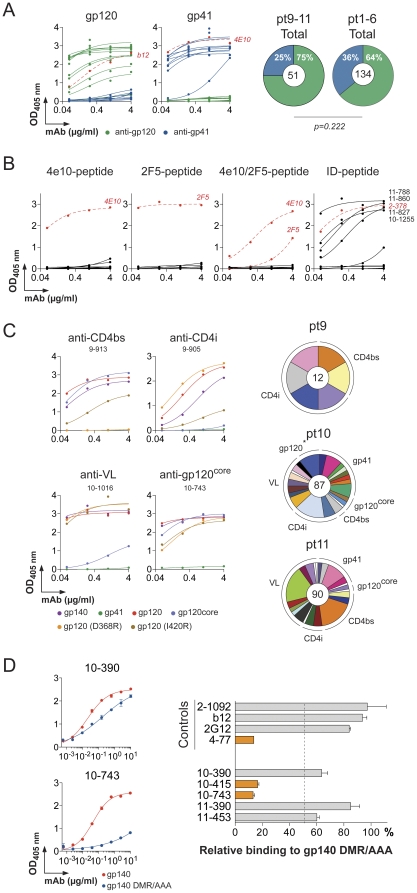
Epitope mapping of anti-gp140 antibodies. **A.** ELISA binding analyses of anti-gp140 antibodies against gp120 and gp41 proteins. Red dotted lines represent the positive antibody controls b12 and 4e10 for gp120 and gp41, respectively [27], [32]. The pie charts show the distribution of gp120 (green slice) and gp41 (blue slice) reactive antibodies among anti-gp140 antibodies isolated from clade A and B HIV-infected patients. Gp120/gp41 reactivities of previously published anti-gp140 antibodies (from pt1 to pt6 HIV patients) are shown for comparison [25]. The total number of tested antibodies is indicated in the center of each pie chart. 2×2 Fisher' exact test shows no statistical difference. **B.** ELISA testing of anti-gp41 antibody binding to MPER-peptides (including 4e10- and 2F5-epitopes) and immunodominant (ID) peptide (9 gp41-reactive antibodies tested). ELISA binding curves of positive antibody controls (4e10, 2F5 and 2-378 [25], [31], [32]) are shown in each graph as red dotted lines. **C.** Antibody binding to gp140, gp120, gp41, gp120^core^, gp120(D368R) and gp120(I420R) proteins measured by ELISA for representative antibodies directed against the CD4 binding site (CD4bs), the CD4 induced site (CD4i), the variable loops (VL) and the core of gp120 (gp120^core^). The distribution of the antibody reactivities to the gp41, CD4bs, CD4i, VL and gp120^core^ according to the clonally related B-cell expansions is depicted for each HIV patient as pie charts. The total number of memory B-cell antibodies is indicated in the center, each pie slice represents a clonal family and the area of the slice is proportional to the number of clonal relatives. Each clonal family is represented by the same color and unique antibodies that are not members of a clonal family are not colored. **D.** Antibody binding to BaL gp140 and gp140(DMR/AAA) mutant protein [37] by ELISA for the representative anti-gp120^core^ antibodies. 10-390 and 10-743 are DMR/AAA-mutation non-sensitive and sensitive, respectively. The bar diagram on the right-hand side shows the relative binding of the anti-gp120^core^ and control antibodies [37] to the gp140(DMR/AAA) mutant compared to the gp140 protein. Error bars indicate the SEM. The orange bars show antibodies sensitive to DMR/AAA-mutation. The cutoff is shown by the dotted line. All the experiments were performed at least in duplicate. Representative data are shown.

To determine whether the anti-gp140 antibodies bound to gp120 or gp41 we performed ELISA experiments with purified proteins. We found that the majority of the antibodies were specific for gp120 protein (75% *vs* 25% for gp41-reactivity) (Figure 3A). None of the anti-gp41 antibodies (n = 13) were directed against the membrane proximal peptides recognized by 2F5 and 4E10 bNAbs [31], [33], and only 31% of the anti-gp41 antibodies showed binding to the immunodominant region of gp41 [36], [42] (Figure 3B).

To further map the fine specificity of the anti-gp120 antibodies we performed ELISAs using mutant proteins: gp120(D368R) interferes with the binding of antibodies to the CD4 binding site (CD4bs) [6]; gp120(I420R) interferes with the binding of antibodies to the CD4 induced co-receptor binding site (CD4i) [43]; gp120^core^ protein lacks variable loops (VL) which interferes with the binding of anti-VL and anti-CD4i antibodies [44]. Thus, antibodies that bind to gp120, gp120(D368R) and gp120(I420R) but not to gp120^core^ were classified as anti-VL antibodies and those that bind to gp120 and gp120(D368R) but not to gp120(I420R) and gp120^core^ were classified as anti-CD4i antibodies. Anti-CD4bs antibodies only show inhibition of binding to gp120(D368R). Finally, antibodies that bind to gp120, gp120(D368R), gp120(I420R) and gp120^core^ were classified as anti-gp120^core^ antibodies. Among these, antibodies that show inhibition of binding to D474A/M475A/R476A gp140 mutant protein (gp140(DMR/AAA)) were classified as anti-CD4bs/DMR antibodies [37].

Similar to the previously characterized elite controller group [25], the anti-gp120 antibodies cloned from pt9-11 targeted all known gp120 epitopes with variable relative representation in all three patients (Figure 3C and Table S2). However, in contrast to the elite controllers and slow progressors, only two of the five antibodies to the gp120^core^ epitope were sensitive to the D474A/M475A/R476A mutation at the outer domain/inner domain junction of gp120, which is essential for optimal viral infectivity [37] (Figures 3D and S2). Although this is a small number of “core” antibodies, the results suggest the existence of additional epitopes that differ from those recognized by conventional anti-CD4bs and anti-CD4bs/DMR antibodies.

### Heterogeneous neutralizing activity

**Figure 4:**
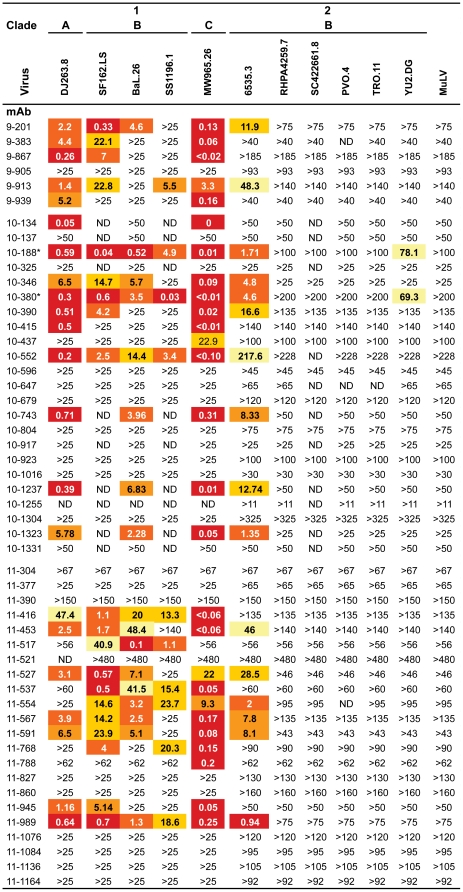
Neutralizing activity of anti-gp140 antibodies in TZM-bl assay. Numbers indicate antibody IgG concentrations in µg/ml to reach the IC50 in the *in vitro* TZM-bl neutralization assay. > indicates that the IC_50_ for a given virus was not reached at the concentration tested. 9-1487 antibody was not tested for viral neutralization. *10-188 and 10-380 are clonally related antibodies. ND, not determined. MuLV, Murine leukemia virus, is the negative control.

**Figure 5:**
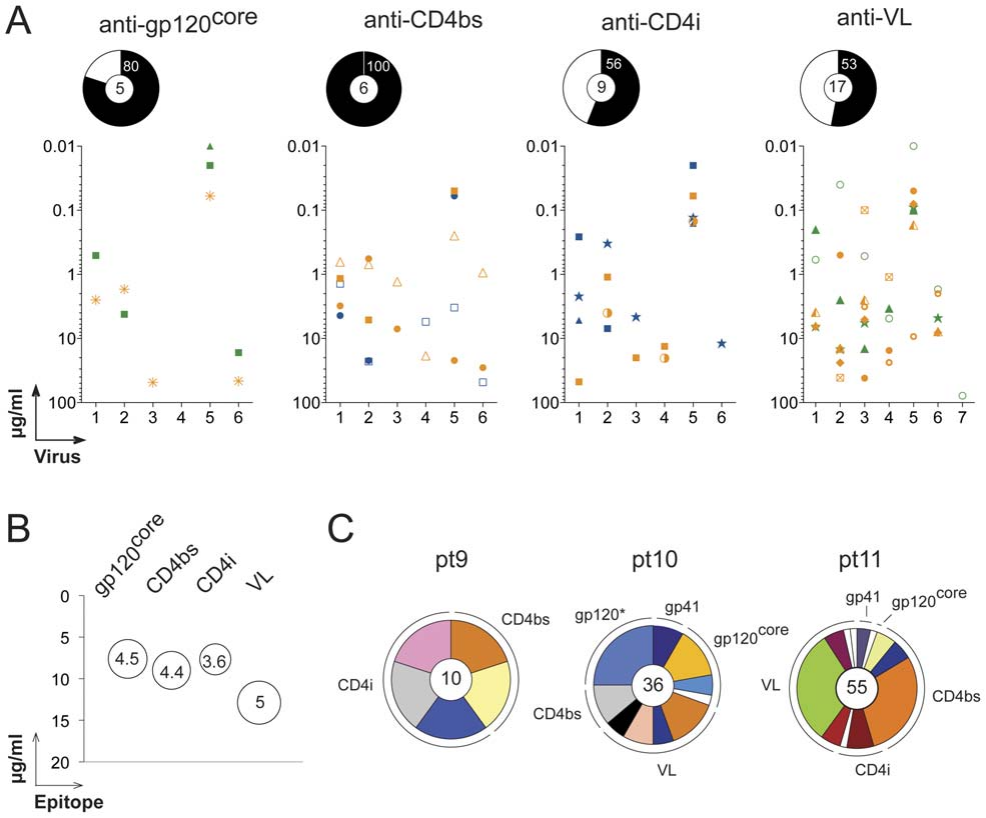
Neutralizing potency and breadth of anti-gp140 antibodies. **A.** The neutralizing activity measured by TZM-bl assay is shown for individual antibodies according to their epitopes (targeted regions of gp120) are shown in the graphs. The *y*-axis shows the IgG antibody concentration required to achieve IC_50_ for each viruses indicated on the *x*-axis: 1, DJ263.8; 2, SF162.LS; 3, BaL26; 4, SS1196.26; 5, MW965.26; 6, 6535,3; 7, YU2.DG. The pie chart above each graph indicates the frequency of antibody neutralizers in each epitope group, with the total number of antibodies tested in the center. **B.** Antibody neutralizing potency and breadth according to the targeted gp120-epitopes. The bubble plot shows for the antibody neutralizers (with neutralization of at least 2 viruses) grouped by gp120-targetted epitopes (*x*-axis), the mean IgG antibody concentration required to achieve IC_50_ (*y*-axis) and the average number of viruses neutralized (indicated by the bubble size and by the number in the center of the bubble). **C.** The distribution of the antibody neutralizers (with neutralization of at least 1 virus) binding to the gp140 epitopes according to the clonally related B-cell expansions is depicted for each HIV patient as pie charts. The total number of neutralizing antibodies is indicated in the center, each pie slice represents a clonal family and the area of the slice is proportional to the number of clonal relatives. Each clonal family is represented by the same color and unique antibodies that are not members of a clonal family are not colored. The star indicates that only one virus out 11 was neutralized by the 2 anti-gp41 antibodies.

To determine the *in vitro* neutralizing activity of the fifty-one anti-gp140 IgG memory antibodies, we measured their capacity to inhibit TZM-bl cell infection by pseudovirus variants [45]. Most of the anti-gp120 antibodies (66%, n = 38) exhibited measurable levels of neutralization activity against our panel of 11 pseudo-viruses (Figure 4). In contrast, only 2 of the 13 anti-gp41 antibodies (10-437 and 11-788) showed any activity and in both cases only against the same tier-1 virus (MW965.26, Figure 4). When segregated according to their gp120 epitope specificity the anti-CD4bs showed the highest frequency of antibody neutralizers followed in order by gp120^core^, CD4i and VL reactive antibodies (Figure 5A). However, the average number of viruses neutralized and the mean IgG concentration required to reach the IC_50_ for neutralization was similar for the different gp120-targeted epitopes (Figure 5B and C).

To determine whether the anti-gp140 antibodies isolated from clade A HIV-infected donors (pt9 and pt10) using clade B YU-2 gp140 bait might display increased neutralization breadth, we measured the neutralizing activity of most of the antibodies from pt9-pt10 patients (n = 19) against a broader panel of 22 additional HIV pseudo-viruses (Table S3). Of the anti-gp120 antibodies that showed neutralizing activity in both virus panels (n = 9, 33 viruses tested in total), tier 1 viruses were most frequently neutralized (67±15% *vs* 9±6% for tier-2 viruses) (Table 1). However, there was no tendency toward preferential neutralization of clade A, B or C viruses by the antibodies derived from clade A HIV patients (Table 1). Significantly, anti-VL 10-188 antibody isolated from the same patient (pt10) was the only one to show activity against the resistant tier-2 YU-2 virus, from which the bait was derived (Figure 4). This antibody ranked in the top 5% of the neutralizers identified in our collection of 185 unique anti-gp140 antibodies 99 of which show some neutralizing activity [25]. We conclude that neutralizing activity of the antibodies derived from clade A infected patients with the clade B YU-2 gp140 trimer are similar in neutralizing activity to those isolated from clade B infected elite controllers and slow progressors [25].

**Table 1:** Neutralization breadth of the selected anti-gp120 antibodies isolated from clade A HIV-infected patients.

	Tier			Clade		
	1	2		A	B	C
**9-201**	83	15		20	31	33
**9-383** #	67	0		*ND*	*ND*	*ND*
**9-867**	50	4		20	6	22
**9-913**	83	11		40	25	22
**9-939**	33	0		20	0	11
**10-188** *	100	19		20	50	22
**10-346** #	67	18		*ND*	*ND*	*ND*
**10-380** *	83	15		20	44	11
**10-390**	67	4		20	19	11
**10-415**	50	7		40	6	22

The numbers correspond to the percentage of virus neutralized.

#not all the viruses were tested, see Table S3.

*10-188 and 10-380 are clonally related antibodies.

*ND*, not determined.

### Neutralizing antibody 10-188 binds to the V3 crown

**Figure 6:**
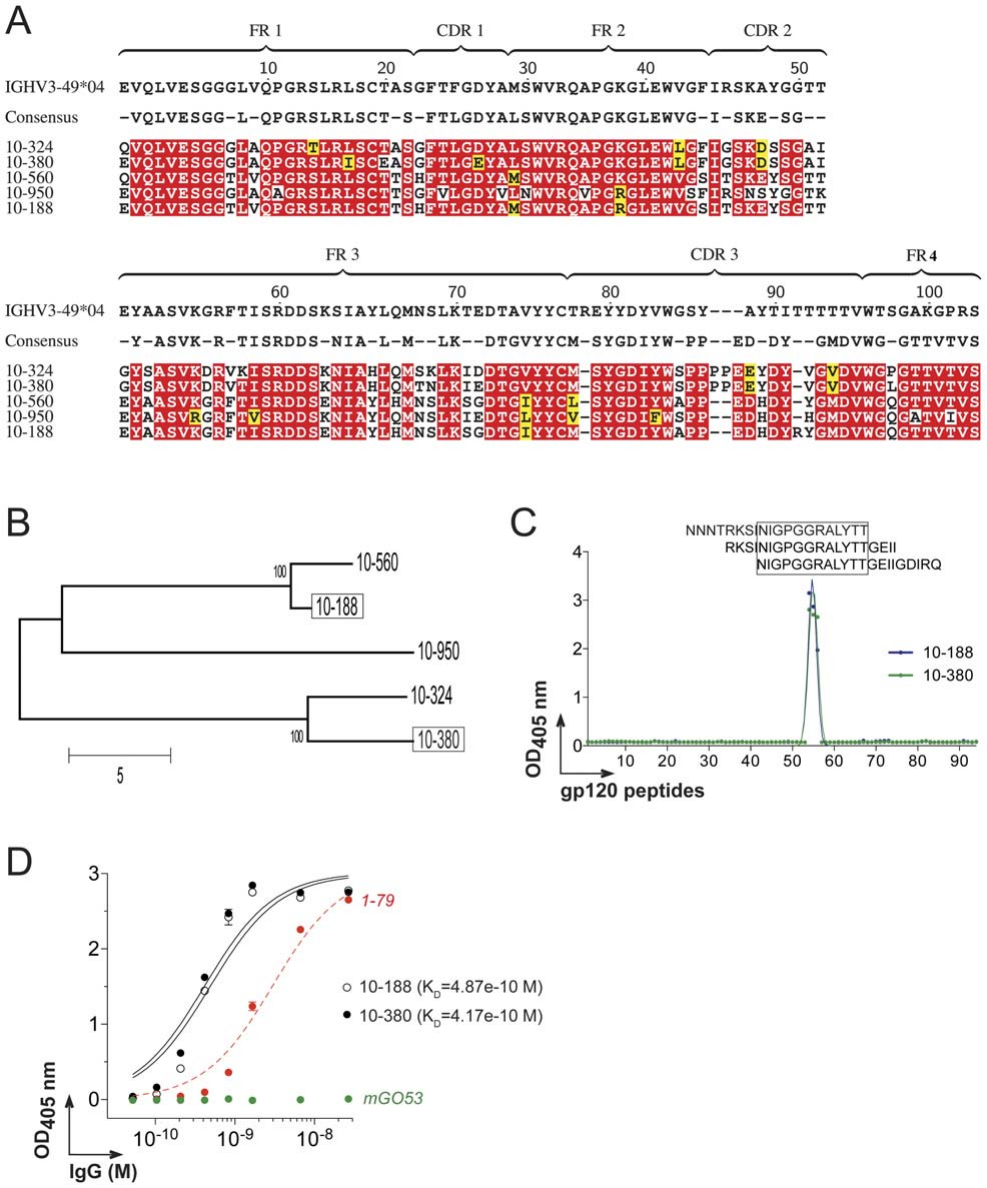
Sequence analyses and epitope mapping of 10-188 antibody. **A.** IgH amino acid alignment of 10-188 with its germline precursor and clonal relatives. Red shading shows amino acid identity, yellow shows biochemical similarity. The consensus sequence is shown above, dashes in this sequence indicate non-conserved residues CDR, complementary determining regions; FR, framework regions. **B.** Phylogenetic tree of the 10-188 clonal family generated from the IgH sequence alignment shown in A. **C.** ELISA graph shows the epitope mapping for 10-188 and 10-380 antibodies using as antigens, overlapping 20-mer peptides covering the entire YU-2 gp120 sequence. The amino acid sequence of each numbered peptide is indicated in Table S4. Sequences of the reactive V3 peptides are indicated on top of the curves. **D.** Graph shows the ELISA measuring the reactivity of 10-188 and 10-380 antibodies against the V3-peptide NNNTRSINIGPGGRALYTT. Green and red lines show the negative (mGO53, [34]) and positive (1–79, [25]) controls, respectively. All the experiments were performed at least in duplicate. Error bars indicate the SEM. Approximate K_D_ values calculated from the ELISA binding curves are indicated.

10-188 was a member of an expanded clonal family containing four other antibody variants characterized by intra-clonal diversity, specifically in the length of their IgH CDR3 regions (Figures 6A and 6B, and Table S2). To determine whether such structural variation could have an influence on neutralization, we assayed the neutralization of 10-380, the most distal clonally related variant of 10-188 (Figures 6A and 6B, and Table S2). 10-380 showed neutralizing potency and breadth comparable to 10-188 when tested for the same virus panels (Figure 4, Tables 1 and S3). To better characterize the epitope recognized by 10-188 and 10-380, we examined their reactivity by ELISA using a library of overlapping 20-mer peptides covering the entire YU-2 gp120 sequence (Figure 6C and Table S4). The epitope targeted by 10-188 and 10-380 was mapped into the crown of the gp120 variable loop 3 (V3), and was composed of the minimal peptide NIGPGGRALYTT (Figures 6C and 6D). Taken together, the results indicate that there is a consensus antibody sequence shared by the different members of the 10-188 clonal family allowing their binding to the V3 crown and potent neutralization.

### Polyreactivity of gp140-specific B cell antibodies

**Figure 7:**
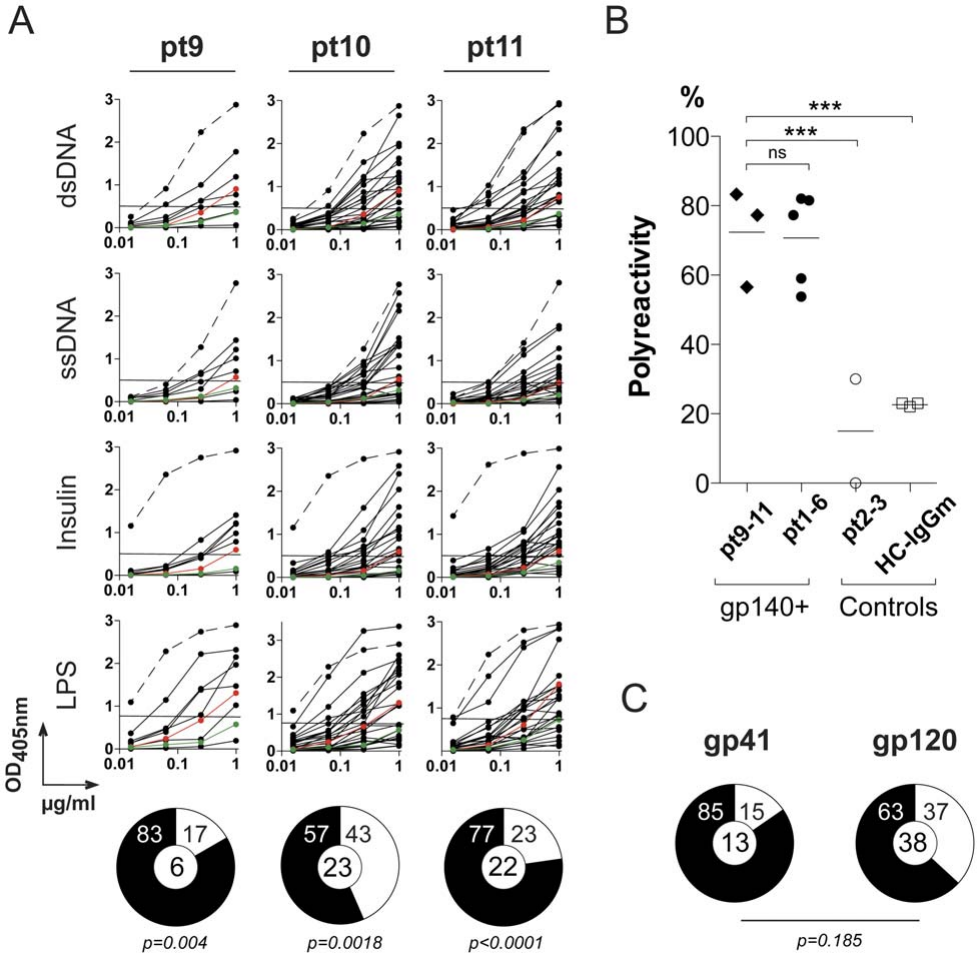
Polyreactivity of anti-gp140 IgG memory B-cell antibodies. **A.** Graphs show ELISAs measuring reactivity against dsDNA, ssDNA, insulin and LPS for IgG antibodies cloned from gp140+ memory B cells from patient pt9, pt10 and pt11. Dotted lines represent the positive control antibody ED38 [61]. Horizontal lines show cut-off OD_405 nm_ for positive reactivity. Green and red lines show the negative control antibody mGO53 and low positive control antibody eiJB40, respectively [34]. All the experiments were performed at least in duplicate. Representative data are shown. Pie charts summarize the frequency of polyreactive (black) and non-polyreactive (white) gp140-reactive memory B cell clones. The number of tested antibodies is indicated in the pie chart center. **B.** The dot plot shows the polyreactivity frequency of gp140-specific IgG memory B-cell antibodies isolated from pt9-pt11 HIV-patients compared to anti-gp140 antibodies from previous studied patients (pt1 to pt6) [25] and control antibodies (non gp140-binding antibodies from pt2-3 patients [25] and HC-IgGm antibodies [41]). Each symbol represents a donor. Groups were compared using 2×5 Fisher's Exact test.^ ***^, p<0.001. **C.** Pie charts summarize polyreactivity of anti-gp140 antibodies grouped by antibody specificity for gp41 and gp120. Reactive (black) and non-reactive (white) IgG antibodies, numbers in the center indicate number of antibodies tested. 2×2 Fisher's Exact test shows no statistical difference between both groups.

Seventy five percent of the anti-gp140 memory antibodies cloned from the elite controller group are polyreactive (pt1-6, [38]). To determine whether the anti-HIV antibodies cloned from pt9-11 patients are also polyreactive, we measured their binding to ssDNA, dsDNA, LPS, and insulin by ELISA (Figure 7A). Although there was no increase in serologic polyreactivity between the three HIV patients and healthy controls (Figure S3), 69% of all of the pt9-11 anti-gp140 memory antibodies were polyreactive (Figure 7B, p<0.0001 compared to uninfected controls and antibodies that did not bind to gp140 antibodies from pt2-3 patients [25], [41]). The frequency of polyreactive antibodies varied between patients (from 57 to 83%): however, as a group, pt9-11-antibodies were indistinguishable from those obtained from pt1-6 [25](69% *vs* 75%, *p* = 0.46). In addition, pt9-11 anti-gp41 antibodies were slightly more polyreactive than anti-gp120 (Figure 7C, [38]). We conclude that increased levels of polyreactivity are found in anti-HIV gp140 antibodies derived from clade A and B infected patients.

## Discussion

We have characterized fifty one new human gp140-specific antibodies cloned from YU2 gp140 trimer-binding IgG memory B cells from clade A and B HIV-infected patients with broad neutralizing serologic activity. Our findings extend previous work on anti-gp140 antibodies isolated from elite controllers infected with clade B HIV-1. Notably the pt9-11 antibodies show (i) an IgG gene repertoire that is enriched for VH1 and Igκ light chain genes, as well as long and charged IgH-CDR3s; (ii) the new memory antibodies bind with high affinities to a number of different epitopes on both gp120 and gp41 proteins; (iii) the majority of the anti-gp120 antibodies show neutralizing activity against tier-1 viruses, while neutralization by anti-gp41 antibodies was rare; (iv) finally, the majority of the anti-gp140 antibodies were polyreactive [25], [38].

Using a clade B gp140 protein as “bait” to isolate memory B cells from clade A HIV patients might be expected to select for cross-reactive B-cell clones with broad neutralizing capacities. Surprisingly, the anti-gp140 antibodies isolated from the clade A HIV-infected donors were indistinguishable from those isolated from the clade B patient pt11 or from the previously studied clade B-infected donors in nearly all respects [25]. How this might change if we were to use clade A envelope proteins remains to be determined. In addition, none of the individual antibodies isolated had bNAb properties. This raises the question of a potential lack of efficiency of our single-cell capture method using the soluble recombinant YU-2 gp140 trimer to isolate broadly neutralizing B cells/antibodies. In contrast to our results, an extremely potent and broad antibody, VRC01, was obtained using an artificially resurfaced protein as bait suggesting that the YU-2 gp140 trimer bait is limiting for antibody capture [30]. However, the two experiments were done with independently collected samples and other factors that might influence the efficiency of cloning such as the primer set and its susceptibility to hypermutation of anti-HIV antibodies were not explored.

It is important to note that two anti-V3 clonally-related antibodies with potent neutralizing activities isolated from clade A HIV pt10 patient, ranked in the top 5% of the antibody neutralizers in our collection of 185 anti-gp140 antibodies. The neutralization spectra of those two antibodies were indeed comparable to the ones described for other V3-specific antibodies that mostly neutralize tier 1-viruses and only 10–25% of tier 2-viruses [26], [46], [47].

Two groups have identified bNAbs from non-clade B HIV-1 infected individuals by EBV-mediated B-cell immortalization and large-scale neutralization screening [26], [29]. Corti *et al.* isolated anti-core-DMR antibody HJ16 from a clade C-infected patient [26], and Walker *et al.* identified anti-VL1/2 antibodies PG9 and PG16 from pt9 [29]. Furthermore, Walker *et al.* recently showed that most of the serum neutralizing activity from pt9 could not be immunoadsorbed with the artificial YU-2 gp140 trimer used in our experiments [48]. This may explain why (i) serum IgGs isolated from pt9 only neutralized YU-2 at very high concentrations (>0.5 mg/ml) and showed a weaker binding reactivity to YU-2 gp140 by ELISA compared to the other serum IgGs; (ii) the relatively low number of gp140-binding cells detected by flow cytometry using the YU-2 gp140 protein ”bait”, (iii) the relatively small number of anti-gp140 antibodies cloned from this donor [29]. Thus, the YU-2 gp140 trimer would not be an appropriate bait to try to capture specific broadly neutralizing antibodies from patients like the one that produced PG9/16.

Two conserved features of anti-HIV-1 memory antibodies stand out: high rates of hypermutation and polyreactivity [25], [38]. We first noted abnormally high levels of somatic hypermutation in anti-HIV-1 antibodies in elite controllers [25]. Reversion of 20 randomly selected antibodies to the germline showed that hypermutation is essential for viral neutralizing activity and breadth [38]. Similarly, reversion of the hypermutations in the recently reported bNAb VRC01 eliminates its neutralizing activity [49]. The fact that high levels of hypermutation were found in antibodies cloned from pt9-11 indicates that this is an important conserved feature of the human anti-HIV-1 antibody response.

Anti-HIV antibody polyreactivity was first noted in an anti-p24 antibody [50]. Haynes *et al.* and others then showed that two anti-gp41 bNAbs, 2F5 and 4E10, were polyspecific and reactive against membrane phospholipid, *i.e.*, cardiolipin [51], [52], [53]. We recently found that this is a more general feature of the human anti-HIV-1 response in clade B infected elite controllers where 75% of the 134 unique anti-gp140 antibodies tested were polyreactive. The present study confirms and extends these findings to clade-A patients, who are not elite controllers. Remarkably, this atypical property of anti-HIV-1 antibodies increases their binding affinity to HIV-1 by allowing a bivalent heteroligation between one high-affinity anti-gp140 combining site and a second low-affinity site on a yet-to-be-defined HIV-1 virus surface component [38]. Enhanced antibody binding by polyreactive heteroligation is reminiscent of the finding that 2F5 and 4E10 bind to epitopes in the membrane proximal region of gp41 but also interact with lipids on the virion membrane [52], [54], [55], [56]. Moreover, Diskin*et al.* recently demonstrated that the anti-CD4i 21c antibody interacts simultaneously with CD4i and CD4 [57]. It has been proposed that heteroligation is positively selected because the number of viral spikes on HIV-1 is too small to allow for bivalent homotypic antibody binding. Although we have not explored the ability of the antibodies described here to mediate heteroligation or to interact with two different parts of the HIV spike as suggested by Diskin *et al.*, conservation of polyreactivity among antibodies from patients infected with different HIV-1 clades further supports the idea that heteroligation may be an important feature of the human anti-HIV-1 antibody response.

## Materials and Methods

### Patients

The study was performed in accordance with and after ethic approval from the Institutional Review Board of the Rockefeller University and of the International AIDS Vaccine Initiative, IAVI (Protocol G) [7]. All patients gave written consent to participate in this study. Peripheral blood mononuclear cells (PBMC) and sera were collected from a cohort of HIV-1 infected individuals (IAVI Protocol G) as previously described [7].

### Anti-gp140 control mAbs

All human anti-gp140 monoclonal antibodies used as controls in this study were previously characterized [25], [27], [28], [31], [32], [37], and expressed by cotransfection of HEK 293T cells as described below.

### Protein production and purification

Expressing vectors encoding for YU-2 gp120 (obtained from J. Sodroski), gp120 (D368R) and gp120 (I420R) mutant proteins (obtained from J. R. Mascola and R. T. Wyatt, [6], [43]), BaL gp140 protein and BaL gp140 (D474A/M475A/R476A) mutant [37] were used to transfect HEK 293T cells as described below. After harvesting of culture supernatants, proteins were purified by successive lectin and nickel-chelating affinity chromatographies [37]. Purified YU-2 gp120^core^ protein [44] was kindly provided by J. R. Mascola and R. T. Wyatt. Gp41 (ectodomain amino acids 541 to 682; strain HxB2) was purchased at Acris, Herford.

### gp140-specific B cell sorting

Peripheral B cells were isolated from patient PBMC by magnetic B cell enrichment using human CD20 microbeads or a B cell enrichment kit (Miltenyi). Single gp140+CD19+IgG+ B cells were identified by staining with biotinylated gp140 [35]/Streptavidin-PE (Caltag), anti-CD19-FITC and anti-IgG-APC (BD Biosciences Pharmingen) and sorted into 96-well PCR plates using a FACSVantage sorter (Becton Dickinson) as previously described [34], [35].

### RT-PCR and expression-vector cloning

Single-cell cDNA synthesis using SuperScript III reverse transcriptase (Invitrogen) followed by nested-PCR amplifications of IgH, Igκ and Igλ genes were performed as previously described [40]. All PCR products were sequenced and analyzed for Ig gene usage, CDR3 analyses and number of VH/Vκ somatic hypermutations (IgBLAST; http://www.ncbi.nlm.nih.gov/igblast and IMGT®; http://www.imgt.org). Control data from previously published anti-gp140 and non gp140-reactive antibodies isolated from 6 clade B HIV-infected patients [25], and antibodies isolated from the IgG+ memory B cells of three healthy donors were used as comparison [41]. Purified digested PCR products were cloned into human Igγ_1_-, Igκ- or Igλ-expressing vectors as previously described [40]. Vectors containing IgH and IgL genes were isolated from transformed-DH10β bacteria using plasmid DNA purification kits (NucleoSpin®Plasmid, Macherey-Nagel; or PureLink™ plasmid maxiprep kit, Invitrogen), then sequenced and compared to the original PCR-product sequences. The memory phenotype of the isolated gp140-specific B cells was confirmed by the presence of gene features associated with post-germinal center cells [58], [59].

### Antibody production and purification

Antibodies were produced by transient co-transfection of exponentially growing HEK 293T cells (ATCC, CRL-11268) using polyethylenimine (PEI)-precipitation method. Briefly, 293T cells growing in DMEM (Gibco® Invitrogen) supplemented with 10% FBS (HyClone, Thermoscientific), and 1 mM sodium pyruvate (Gibco® Invitrogen), antibotics/antimycotics (Gibco® Invitrogen) were washed at 80% cell-confluency using serum-free DMEM for 1 h. The medium was then replaced with 20 ml of DMEM supplemented with 1% Nutridoma-SP (Roche), 1 mM sodium pyruvate (Gibco® Invitrogen), and antibotics/antimycotics (Gibco® Invitrogen). Equal amounts of Ig-H and IgL-expressing vectors (15 µg of each plasmid DNA per plate) were mixed in 1.2 ml of 150 mM NaCl. After adding 200 µl of 0.45 mg/ml PEI (Sigma) and vortexing for 20 s, the mixture was incubated for 10 min at room temperature to allow formation of precipitates and gently distributed to the culture plate. Cells were cultivated 4 days at 37°C in a 5% CO_2_ air atmosphere before harvesting of the supernatants. IgG antibodies were affinity purified using Protein G sepharose beads (GE Healthcare) according to the manufacturer's instructions and their concentration determined by IgG-ELISA as previously described [34].

### ELISAs

The antibody binding to YU-2 gp140 protein was tested by ELISA as previously described [25]. Briefly, high-binding 96-well ELISA plates (Costar) were coated overnight with 100 ng/well of purified gp140 in PBS. After washings, plates were blocked 2 h with 2% BSA, 1 µM EDTA, 0.05% Tween-PBS (Blocking buffer). gp140-coated plates were incubated 2 h with IgG antibodies diluted at 4 µg/ml and three consecutive 1∶4 dilutions in PBS. After washings, the plates were revealed by incubation for 1 h with goat HRP-conjugated anti-human IgG (Jackson ImmunoReseach) (at 0.8 µg/ml in blocking buffer) and by adding 100 µl of HRP chromogenic substrate (ABTS solution, Invitrogen) after washing steps. Optical densities were measured at 405 nm (OD_405 nm_) using an ELISA microplate reader (Molecular Devices). Background values given by incubation of PBS alone in coated wells were subtracted. For epitope mapping analyses, the anti-gp140 antibodies were tested by ELISAs as described above using as antigens, YU-2 gp120, gp41, gp120^core^, gp120 D368R, and gp120 I420R proteins as antigens. To assay the antibody binding to gp41 peptides (Imunodominant region (ID), DQQLLGIWGCSGKLICTTTV; 2F5, SQNQQEKNEQELLALDKWAS; 4E10, LWNWFDITKWLWYIKIFIMI; 2F5-4E10, ELLALDKWASLWNWFDITKW) [36] and to YU-2 gp120 overlapping peptides (Table S4), the anti-gp140 IgG antibodies were tested using a previously described peptide-ELISA method [60]. Analysis of the antibody binding to BaL gp140 and gp140 (DMR/AAA) mutant protein was performed using a previously described capture-ELISA assay [37]. The relative binding of anti-gp120^core^ antibodies to gp140 (DMR/AAA) mutant protein was calculated with OD values within the linear range of the ELISA curves, using the following formula: (OD^gp140 (DMR/AAA)^/OD^gp140^) x 100. Antibodies and serum IgGs were tested for polyreactivity as previously described [34], [38] and considered polyreactive when they recognized at least two structurally different antigens out of the four tested; ssDNA, dsDNA, insulin, and LPS. Threshold values for reactivity were determined by using control antibodies mGO53 (negative), eiJB40 (low positive), and ED38 (high positive) [34], [61]. IgGs isolated from the serum of HIV, and SLE patients and healthy humans [38] by Protein G affinity purification were tested by polyreactivity-ELISA as described above. All ELISA experiments were performed at least in duplicates.

### Surface plasmon resonance

All experiments were performed with a Biacore T100 (Biacore, Inc) in HBS-EP+ running buffer (Biacore, Inc) at 25°C. YU-2 gp140 and gp120 proteins at 125 µg/ml were immobilized on CM5 chips (Biacore, Inc.) by amine coupling at pH 4.5 resulting in an immobilization level of 10,000 RUs. For kinetic measurements on the gp140- and gp120-derivatized chips, IgGs were injected through flow cells at 40 nM and 4 successive 1∶2-dilutions in HBS-EP+ running buffer (Biacore, Inc.) at flow rates of 40 µl/min with 3 min association and 5 min dissociation. The sensor surface was regenerated between each experiment with a 30 second injection of 10 mM glycine-HCl pH 2.5 at a flow rate of 50 µl/min. Off rate (k_d_ (s^−1^)), on rate (*k*_a_ (M^−1^ s^−1^ and RU^−1^ s^−1^ for *k*_a2_)) and binding constants (*K*_D_ (M) or *K*_A_ (M^−1^ and RU^−1^ for *K*_A2_)) were calculated after subtraction of backgrounds (binding to control flow cells and signal of the HBS-EP+ running buffer) using Biacore T100 Evaluation software using the kinetic analysis and the bivalent binding model. The *K*_A1_ value was used as an estimation of apparent *K*_A_ (*K*_A_^app^) as previously described [62]. The sensorgrams showed in Figures 1E and S1 are derived from the Biacore data processing using Scrubber 2 sofware (Center for Biomolecular Interaction Analysis, University of Utah).

### Neutralization assays

Virus neutralization was measured using a luciferase-based assay in TZM.bl cells as previously described [45]. Antibodies were also tested for virus neutralization at Monogram Biosciences [63].

### Multiple sequence alignments

All multiple sequence alignments were conducted using CLUSTALW2 with default parameters. Alignment shadings were generated using TeXshade package. The consensus sequences for multiple alignments were generated based on identity and similarity between residues (≥70%). The amino acids were grouped based on biochemical similarity as: FYW, ILVM, RK, DE, GA, ST and NQ. The relationship between sequences was generated using the Neighbor-Joining method. The bootstrap consensus tree inferred from 1,000 replicates was taken to represent the relationship. The percentage of replicate trees in which the associated sequence clustered together in the bootstrap test (1,000 replicates) is shown next to the branches. The tree is drawn to scale, with branch lengths in the same units as those of the evolutionary distances used to infer the phylogenetic tree. The evolutionary distances were computed using the number of differences method and are in the units of the number of amino acid differences per sequence. All ambiguous positions were removed for each sequence pair. Evolutionary analyses were conducted in MEGA5 (http://www.megasoftware.net).

### Statistics

*P* values for Ig gene repertoire analyses, analysis of lengths, positive charges, of IgH CDR3, and antibody reactivity were calculated by 2×2 or 2×5 Fisher's Exact test. The numbers of VH and Vκ mutations were compared across groups of antibodies using unpaired student t-test.

## Supporting Information

Figure S1 **Binding affinity of anti-gp120 antibodies isolated from clade A HIV-infected patients.** Surface plasmon resonance (SPR) analyses of the interaction of the selected anti-gp140/gp120 IgG antibodies with the gp140 (**A**) and gp120 (**B**) ligands immobilized on the sensor chips. Graphs show SPR sensorgrams over time for the binding of the selected antibodies. RU; response units. The on-rate, off-rate and affinity constant values for the antibody/ligand interactions shown in A and B are given the table in **C**. *10-188 and 10-380 are clonally-related antibodies. M, mol/; s, seconds. -, not determined.
(PDF)

Figure S2 **Reactivity of anti-gp120core antibodies against gp140 DMR/AAA mutant.** ELISA binding curves show the reactivity of anti-gp120^core^ antibodies against BaL gp140 and BaL gp140 DMR/AAA mutant [37]. Antibodies sensitive (anti-gp120^core^, 4-77 antibody) and non-sensitive (anti-VL 2-1092, b12 and 2G12 antibodies) to DMR/AAA triple mutation were used as controls [37]. Mean values from two independent experiments are shown. Error bars indicate SEM.
(PDF)

Figure S3 **Reactivity of serum IgG from HIV patients.** Serum IgG reactivity of HIV patients pt9 to pt11 (red lines) and three healthy donors used as controls (blue lines) against dsDNA, ssDNA, Insulin, and LPS used as antigens in the polyreactivity ELISA [34], [38]. The green line shows the reactivity of serum IgG from one SLE patient used as positive control [64].
(PDF)

Table S1 **Neutralizing activity of purified IgG from HIV patient sera in TZM-bl assay.** Numbers indicate serum IgG concentrations in µg/ml to reach the IC_50_ in the TZM-bl neutralization assay. > indicates that the IC_50_ for a given virus was not reached at the concentration tested. ND, not determined.
(PDF)

Table S2 **Repertoire and reactivity of gp140-specific antibodies.** *10-188 and 10-380 are clonally related antibodies. (-) and (+) indicate the numbers of negatively and positively charged amminoacids in the IgH complementary determining region (CDR3), respectively. VHmut and Vk/lmut indicate the total number of mutations in the VH and VL *Ig* genes. # exp., number of clonally related expansions; # rel., number of conally related members. gp41-ID, gp41 immunodominant epitope; V3, variable loop 3 of gp120. Neut., neutralization activity; Poly., polyreactivity.
(PDF)

Table S3 ***In vitro*** **neutralization assay of the anti-HIV gp140 antibodies isolated from clade A HIV-infected donors.** Neutralization testing was performed by Monogram Biosciences using single round of replication pseudovirus assay [59]. Numbers indicate antibody IgG concentrations in µg/ml to reach the IC_50_ in the neutralization assay. > indicates that the IC_50_ for a given virus was not reached at the concentration tested. ND, not determined. aMLV is the negative control of the assay. *10-188 and 10-380 are clonally related antibodies.
(PDF)

Table S4 **Sequence of YU-2 gp120 overlapping peptides.**
(PDF)
