# Supplementary material for: Memory B Cell Antibodies to HIV-1 gp140 Cloned from Individuals Infected with Clade A and B Viruses
Source: PLoS One. 2011 Sep 8;6(9):e24078. doi: 10.1371/journal.pone.0024078 (PMC3169578; doi:10.1371/journal.pone.0024078)
Supplement: Figure S1 — Binding affinity of anti-gp120 antibodies isolated from clade A HIV-infected patients. Surface plasmon resonance (SPR) analyses of the interaction of the selected anti-gp140/gp120 IgG antibodies with the gp140 (A) and gp120 (B) ligands immobilized on the sensor chips. Graphs show SPR sensorgrams over time for the binding of the selected antibodies. RU; response units. The on-rate, off-rate and affinity constant values for the antibody/ligand interactions shown in A and B are given the table in C. *10-188 and 10-380 are clonally-related antibodies. M, mol/; s, seconds. -, not determined. (PDF) [file pone.0024078.s001.pdf]

A

B

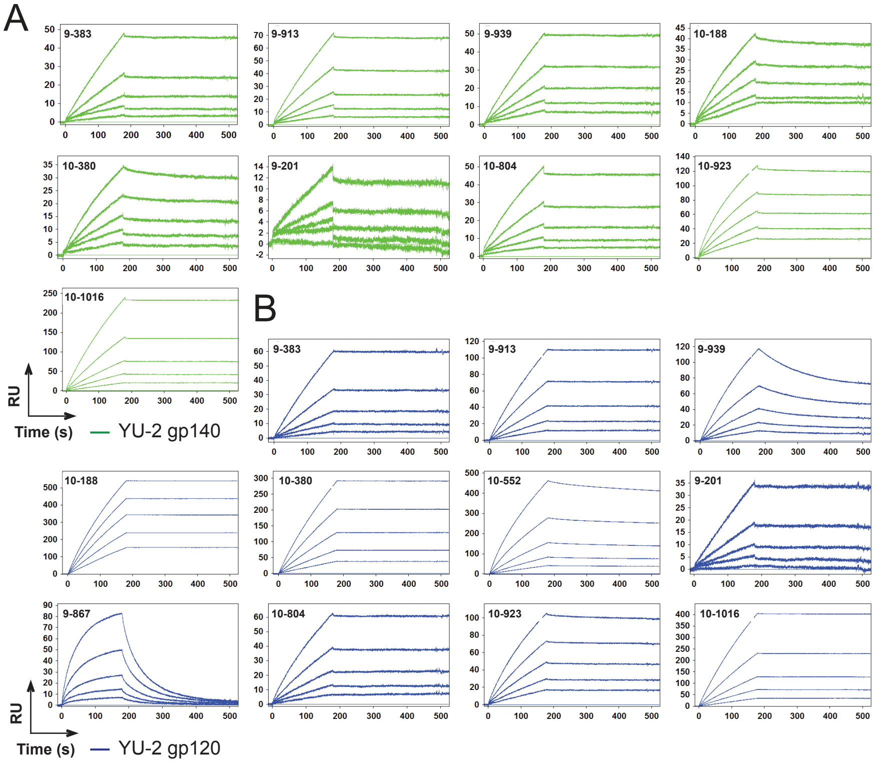

C

	gp120			gp140		
	k_{a1} ($M^{-1}s^{-1}$)	k_{d1} (s^{-1})	K_{A1} (M^{-1})	k_{a1} ($M^{-1}s^{-1}$)	k_{d1} (s^{-1})	K_{A1} (M^{-1})
**9-201**	9.4E+03	3.6E-05	**2.6E+08**	7.2E+03	1.5E-04	**4.9E+07**
**9-383**	3.5E+04	2.1E-05	**1.7E+09**	2.0E+04	7.0E-05	**2.9E+08**
**9-867**	6.3E+05	4.3E-02	**1.5E+07**	-	-	-
**9-913**	5.4E+04	3.7E-05	**1.5E+09**	3.9E+04	4.8E-04	**8.1E+07**
**9-939**	1.4E+05	5.9E-03	**2.3E+07**	3.5E+05	1.1E-05	**3.1E+10**
**10-188***	8.8E+05	9.4E-06	**9.4E+10**	2.8E+06	1.3E-03	**2.2E+09**
**10-380***	1.0E+05	1.7E-06	**6.0E+10**	5.7E+05	2.1E-03	**2.7E+08**
**10-552**	5.2E+04	4.6E-04	**1.1E+08**	-	-	-
**10-804**	6.6E+04	1.4E-06	**4.8E+10**	5.7E+04	5.0E-05	**1.1E+09**
**10-923**	4.0E+06	1.7E-03	**2.4E+09**	4.8E+05	6.5E-06	**7.5E+10**
**10-1016**	4.9E+05	6.3E-06	**7.7E+10**	3.5E+05	4.8E-07	**7.3E+11**
