# Supplementary figures and images for: Memory B Cell Antibodies to HIV-1 gp140 Cloned from Individuals Infected with Clade A and B Viruses
Source: PLoS One. 2011 Sep 8;6(9):e24078. doi: 10.1371/journal.pone.0024078 (PMC3169578; doi:10.1371/journal.pone.0024078)

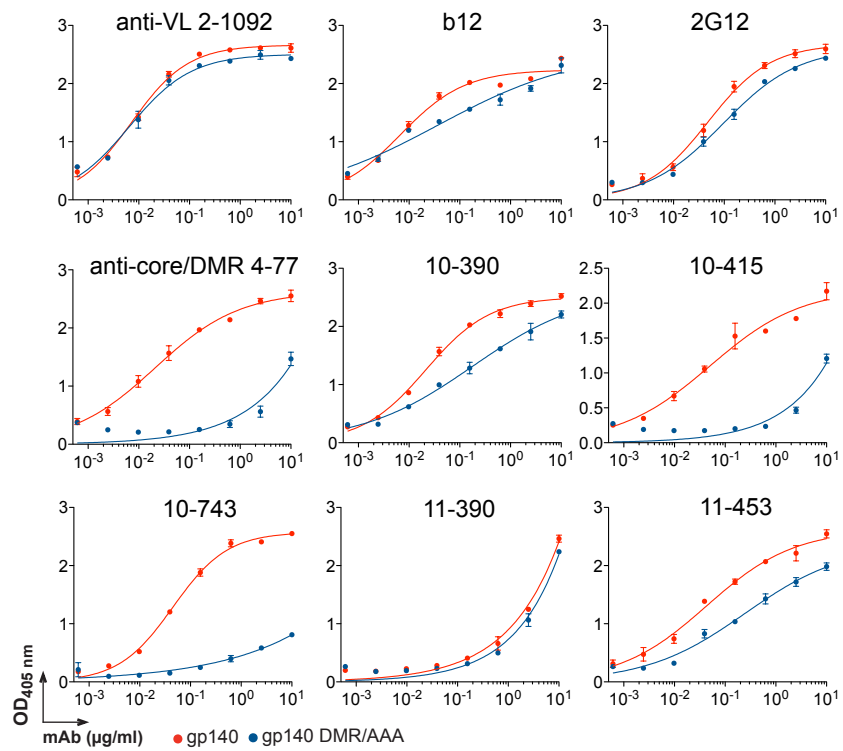

Supplement: Figure S2 — Reactivity of anti-gp120core antibodies against gp140 DMR/AAA mutant. ELISA binding curves show the reactivity of anti-gp120core antibodies against BaL gp140 and BaL gp140 DMR/AAA mutant [37]. Antibodies sensitive (anti-gp120core, 4-77 antibody) and non-sensitive (anti-VL 2-1092, b12 and 2G12 antibodies) to DMR/AAA triple mutation were used as controls [37]. Mean values from two independent experiments are shown. Error bars indicate SEM. (PDF) [file pone.0024078.s002.pdf]

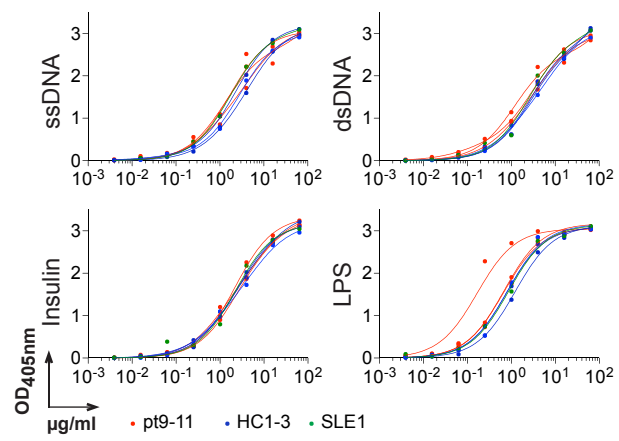

Supplement: Figure S3 — Reactivity of serum IgG from HIV patients. Serum IgG reactivity of HIV patients pt9 to pt11 (red lines) and three healthy donors used as controls (blue lines) against dsDNA, ssDNA, Insulin, and LPS used as antigens in the polyreactivity ELISA [34], [38]. The green line shows the reactivity of serum IgG from one SLE patient used as positive control [64]. (PDF) [file pone.0024078.s003.pdf]
