# Supplementary material for: Memory B Cell Antibodies to HIV-1 gp140 Cloned from Individuals Infected with Clade A and B Viruses
Source: PLoS One. 2011 Sep 8;6(9):e24078. doi: 10.1371/journal.pone.0024078 (PMC3169578; doi:10.1371/journal.pone.0024078)
Supplement: Table S1 — Neutralizing activity of purified IgG from HIV patient sera in TZM-bl assay. Numbers indicate serum IgG concentrations in µg/ml to reach the IC50 in the TZM-bl neutralization assay. > indicates that the IC50 for a given virus was not reached at the concentration tested. ND, not determined. (PDF) [file pone.0024078.s004.pdf]

Tier	Clade	Strain	pt9	pt10	pt11
1	A	DJ263.8	20.6	29	33.3
	B	SF162.LS	21.9	3.7	1.8
		SS1196.1	>500	9.5	73.1
		BaL.26	95.7	16.4	5.1
	C	MW965.26	0.4	0.8	2.3
2	B	6535.3	>500	7.3	9.2
		RHPA4259.7	>500	18.6	15.6
		SC422661.8	>500	52.1	55.6
		TRO.11	>500	14.2	23.5
		PVO.4	>500	89.9	ND
		CAAN5342.A2	ND	ND	17.1
		YU2.DG	554	61	32

<1
1-5
5-10
10-25
>25
µg/ml
