# Supplementary material for: Memory B Cell Antibodies to HIV-1 gp140 Cloned from Individuals Infected with Clade A and B Viruses
Source: PLoS One. 2011 Sep 8;6(9):e24078. doi: 10.1371/journal.pone.0024078 (PMC3169578; doi:10.1371/journal.pone.0024078)
Supplement: Table S2 — Repertoire and reactivity of gp140-specific antibodies. *10-188 and 10-380 are clonally related antibodies. (-) and (+) indicate the numbers of negatively and positively charged amminoacids in the IgH complementary determining region (CDR3), respectively. VHmut and Vk/lmut indicate the total number of mutations in the VH and VL Ig genes. # exp., number of clonally related expansions; # rel., number of conally related members. gp41-ID, gp41 immunodominant epitope; V3, variable loop 3 of gp120. Neut., neutralization activity; Poly., polyreactivity. (PDF) [file pone.0024078.s005.pdf]

pt9

					Heavy chain						Light chain								
mAb name	VH	DH	JH	(-)	CDR3 (aa)	(+)	Length	VHmut	κ/λ	Vκ/λ	Jκ/λ	CDR3	Length	Vκ/λmut	# exp.	# rel.	Epitopes	Neut.	Poly.
9-201	3-7	3-9	3	2	RYRTYYDILTGNAQSHD	3	17	25	k	3-15	2	QQYNSWPPLCT	11	14	2	1	CD4i	X	X
9-383	4-61	3-3	4	1	SGYFWSGDMYYFAH	1	14	36	k	3-20	2	QQHDRSPMPMYT	12	20	2	2	CD4bs	X	X
9-867	4-59	2-2	4	2	LSVTEIPAAVFLDS	0	14	36	k	3-15	1	QQYHDWPWT	9	21	2	1	CD4i	X	/
9-905	4-59	3-9	6	2	VALPYHDLLTGYPKYFYMDV	2	20	28	k	3-20	3	QQYGDFPFT	9	9	2	2	CD4i	/	X
9-913	1-69	6-19	1	3	LSRPPDRWLGTQRAEYLQD	3	19	37	k	1-39	5	QQTYNTRPMT	10	17	2	2	CD4bs	X	X
9-939	1-2	3-10	5	2	DSRMFDS	1	7	30	l	7-43	3	LLYSGGAWV	9	25	2	2	CD4i	X	X

pt10

					Heavy chain						Light chain								
mAb name	VH	DH	JH	(-)	CDR3 (aa)	(+)	Length	VHmut	κ/λ	Vκ/λ	Jκ/λ	CDR3	Length	Vκ/λmut	# exp.	# rel.	Epitopes	Neut.	Poly.
10-134	4-39	2-21	5	4	HWVTDCDGDCFTWLDP	1	16	57	k	4-1	2	QQFYTTPYT	9	35	9	4	gp120	X	/
10-137	1-2	3-16	4	4	SDYDYEWEYYRLAY	1	14	32	k	2-28	1	MQGLQTPWT	9	18	6	5	gp41	/	/
10-1487	3-23	2-8/5-12/-24	4	1	LGHEYRTNYRLFQY	3	14	41	k	1-9	5	QQLLSYPHT	9	21	2	2	gp41	ND	X
10-188*	3-49	3-16	6	5	YGDIYWAPPEDHDYYGMDV	0	19	36	l	1-44	3	AAWDDSLNGPV	11	28	5	5	V3	X	/
10-380*	3-49	3-16	6	5	YGDIYWSPPPPEEYDYVGVDV	0	21	36	l	1-44	3	ATWDASLNRPT	11	30			V3	X	/
10-325	3-48	4-4/6-6/-19	6	3	GPDFYSGRPTPQGRTYDYFMDL	2	22	41	k	1-12	4	QQTNSFPLT	9	39	2	1	gp41	/	X
10-346	1-69	4-4/-11	6	3	GDSGSSSPFDYIYMDV	0	16	41	k	3-15	4	QQYNNWILT	9	9	2	2	VL	X	X
10-390	1-69	3-3	6	2	DGRQIFGASNRSGMDV	2	16	55	k	3-20	1	QQYASSPWA	9	24	5	4	gp120[core]	X	X
10-415	1-18	5-24/3-9/-10	6	3	DGEWQIMNYYYKGMDV	1	16	20	k	3-20	1	QQYGHSPRT	9	12	2	1	CD4bs/DMR	X	X
10-437	1-69	3-3/-9	1	3	DVLNTGWDHEYYQQ	1	14	57	l	2-23	3	CSYVRRI	7	32	3	2	gp41	X	X
10-552	3-43	2-21	6	4	DECNGDCYSPHYYYYMDV	1	18	32	k	3-11	4	QQRHKWPVT	9	18	3	3	VL	X	/
10-596	3-23	3-16	4	1	GPLGRFLDF	0	9	40	l	2-8	1	SSYAGSNTFYV	11	27	5	2	CD4i	/	/
10-647	1-2	3-3	4	0	NVVIPAARPLPRHFVA	3	16	44	l	2-23	3	LSYAGDNTWV	10	35	7	3	gp41	/	X
10-679	1-2	3-3/-9	5	1	ILVIPGARPLPRHFDP	3	16	46	l	2-23	3	CSYVGDDTWV	10	37	2	1	gp41	/	X
10-743	3-43	3-3	6	3	DGASTGKYYDFWGGTSRRGKYYMDV	4	25	38	k	3-20	4	QQYGTSPLT	9	20	1	single	CD4bs/DMR	X	/
10-804	1-2	3-22	4	2	VSYYDNSGSD	0	10	36	k	3-20	3	QHYGSSLLT	9	16	13	5	CD4i	/	X
10-917	4-59	3-3	4	1	GFIFGGVFFDY	0	11	19	k	1-39	2	QQSFRTPYT	9	23	1	single	VL	/	X
10-923	1-69	2-21/4-17	6	3	EVGRDSKHFGLMDV	3	14	51	k	3-15	1	QQYYNRPPWT	10	25	3	3	VL	/	/
10-1016	7-4	6-25	4	0	SSLGY	0	5	29	k	2-24	1	MAVTQNPHRWT	11	28	3	1	VL	/	/
**10-1237**	**1-69**	**3-3**	**5**	**2**	**SRTTIFGVAQDNWFDP**	**1**	**16**	**39**	**k**	**1-39**	**4**	**QQSYSTPLT**	**9**	**19**	**4**	**2**	**CD4bs**	**X**	**/**
**10-1255**	**4-34**	**2-8**	**4**	**2**	**EQGSCSGGFCSSVEV**	**0**	**15**	**58**	**k**	**3-20**	**1**	**QQYASSPRM**	**9**	**31**	**2**	**1**	**gp41-ID**	**/**	**/**
10-1304	4-31	3-3	6	2	GVVGATGYDFFYMDV	0	15	31	k	3-15	4	QQYNMWPT	8	14	2	1	gp41	/	X
**10-1323**	**1-69**	**6-6**	**6**	**3**	**GDSGSSSPFDYIYMDV**	**0**	**16**	**40**	**k**	**3-15**	**4**	**QQYNNWILT**	**9**	**10**	**2**	**2**	**VL**	**X**	**X**
10-1331	1-2	6-6	4	3	DPFVLVDDTTP	0	11	49	k	3-11	4	QHRNNWPRALT	11	13	3	2	VL	/	X

pt11

					Heavy chain						Light chain								
mAb name	VH	DH	JH	(-)	CDR3 (aa)	(+)	Length	VHmut	κ/λ	Vκ/λ	Jκ/λ	CDR3	Length	Vκ/λmut	# exp.	# rel.	Epitopes	Neut.	Poly.
11-304	3-11	3-3/3-9/3-16	4	2	GGFGGNYYVRDHYFDL	2	16	40	k	3-15	1	QQYDKLPGT	9	26	5	2	VL	/	X
11-377	4-34	3-9	4	2	AYLRYSNFVPKSAECDH	3	17	22	k	1-5	2	QQYSSYPYS	9	22	2	1	VL	/	X
11-390	4-59	3-3	5	2	DLTPVAPWSHFPVRNNFDP	2	19	34	l	2-11	3	CSYVDFDTVI	10	24	2	1	gp120core	/	X
11-416	1-69	2-2	4	3	ALISDDCATTICYLPFDS	0	18	24	k	4-1	1	QQYYTTPQT	9	6	1	single	CD4i	X	X
11-453	4-48	3-3	4	0	GSRAWSPNSGPFHY	2	14	27	l	3-21	1	QVWHTSAYV	9	18	1	single	gp120core	X	/
11-517	4-34	3-22	3	6	GSARTLHSDDSAEYYDQEPFDV	2	22	39	k	4-1	1	QQYHTPPWT	9	22	1	single	VL	X	/
11-521	4-31	3-16	3	2	TSMLRESRWAVDV	2	13	25	l	2-8	3	SSYAGTKNLV	10	13	2	1	VL	/	X
11-527	1-69	3-22	6	2	RDRNAYRAYYYYGLDV	3	16	22	k	3-20	4	QQYGRSMT	8	4	3	2	CD4bs	X	/
11-537	1-69	4-17	3	6	ELAWVNQAEGEYGDYLDAFDI	0	21	13	k	1-5	1	QQYNSFPGT	9	7	3	3	VL	X	/
11-554	1-18	6-6/6-25/6-13	3	2	PMVAARGGDAFDV	1	13	32	k	3-20	5	QQYGSSPPLT	10	11	17	2	VL	X	X
11-567	1-69	4-23/4-4	4	1	ARGVMTGARNTYFDL	2	15	35	k	1-5	5	QQYESHFMT	9	19	3	3	VL	X	X
11-591	1-69	7-27/5-24	3	3	DKTLGQQMVRKTGDALDV	3	18	26	k	1-5	2	QQYGSSSPDT	10	20	1	single	VL	X	X
11-768	1-24	3-16	4	2	DRLGRRLGELTAGFDY	3	16	21	k	3-11	4	QQRSYWPPGLT	11	8	4	3	CD4i	X	/
11-788	1-69	3-3	4	3	TDGLCWSPDCRGPFDY	1	16	46	k	1-5	1	QQYHNYVTT	9	15	2	2	gp41-ID	X	X
11-827	1-69	3-3	4	3	RQKYEDMATAQLFDY	2	15	47	k	1-33	2	QHYTNIPQMYT	11	34	3	3	gp41-ID	/	X
11-860	4-61	3-10	4	2	LGPFLLVGEFQYYFDS	0	16	43	k	1-9	5	QQLNSYSIT	9	21	9	4	gp41-ID	/	X
11-945	1-18	3-9	6	2	DVGRILGAPAPIFGGFHSYYGLDV	2	24	22	k	2-29	1	MQSKQLPWT	9	15	3	2	CD4bs	X	X
11-989	1-69	3-10	6	2	RLTVRRGGIIERHYLMDV	5	18	32	k	1-27	3	QKYGSALFT	9	11	16	3	CD4bs	X	X
11-1076	1-2	3-3	5	1	VLGGLNWFDP	0	10	27	l	8-61	3	LLYVGPGISV	10	20	4	2	VL	/	X
11-1084	3-53	1-26	4	2	EKFRVGISKGGWFDH	3	15	27	l	2-23	1	CSYVGSSTYV	10	18	3	2	gp41	/	X
11-1136	4-31	3-22	3	1	IPRVTMIRNAFDT	2	13	36	l	2-8	3	CSYAATYNFWV	11	20	4	2	CD4i	/	X
11-1164	3-23	6-13/6-19	4	3	DEGRSAGTPKRYLDH	4	15	30	k	2-28	2	MQSLQTSYT	9	7	1	single	gp41	/	X
