# Supplementary material for: Memory B Cell Antibodies to HIV-1 gp140 Cloned from Individuals Infected with Clade A and B Viruses
Source: PLoS One. 2011 Sep 8;6(9):e24078. doi: 10.1371/journal.pone.0024078 (PMC3169578; doi:10.1371/journal.pone.0024078)
Supplement: Table S3 — In vitro neutralization assay of the anti-HIV gp140 antibodies isolated from clade A HIV-infected donors. Neutralization testing was performed by Monogram Biosciences using single round of replication pseudovirus assay [59]. Numbers indicate antibody IgG concentrations in µg/ml to reach the IC50 in the neutralization assay. > indicates that the IC50 for a given virus was not reached at the concentration tested. ND, not determined. aMLV is the negative control of the assay. *10-188 and 10-380 are clonally related antibodies. (PDF) [file pone.0024078.s006.pdf]

Clade	Tier	Strain	9-201	9-383	9-867	9-905	9-913	9-939	10-188*	10-346	10-380*	10-390	10-415	10-437	10-552	10-596	10-647	10-679	10-804	10-923	10-1016	10-1304
A	2	92RW020	>50	>50	>50	>50	>50	>50	>50	>50	>50	>50	>50	>50	>50	>50	>50	>37	>50	>50	>50	>50
		93UG077	>50	ND	>50	>50	23.09	>50	>50	ND	>50	>50	24.65	>50	ND	>50	>50	>37	>50	>50	ND	ND
		94UG103	>50	>50	>50	>50	>50	>50	>50	>50	>50	>50	>50	>50	>50	>50	>50	>37	>50	>50	>50	>50
		MGRM-A-010	>50	ND	>50	>50	>50	>50	>50	ND	>50	>50	>50	>50	ND	>50	>50	>37	>50	>50	ND	ND
B	1	NL43	1.34	2.21	>50	>50	0.49	>50	16.7	>50	>50	0.57	1.49	>50	>50	>50	>50	>37	>50	>50	>50	>50
	2	92BR020	20.5	>50	>50	>50	>50	>50	1.79	48.38	1.25	>50	>50	>50	7.38	>50	>50	>50	>50	>50	>50	>50
		APV13	>50	ND	>50	>50	>50	>50	>50	ND	>50	>50	>50	>50	ND	>50	>50	>37	>50	>50	ND	ND
		APV17	>50	ND	>50	>50	>50	>50	>50	ND	>50	>50	>50	>50	ND	>50	>50	>37	>50	>50	ND	ND
		APV6	>50	ND	>50	>50	>50	>50	22.77	ND	13.82	>50	>50	>50	ND	>50	>50	>37	>50	>50	ND	ND
		JRFL	>50	>50	>50	>50	>50	>50	>50	>50	>50	>50	>50	>50	>50	>50	>50	>37	>50	>50	>50	>50
		JRCSF	>50	>50	>50	>50	>50	>50	>50	ND	>50	>50	>50	>50	ND	>50	>50	>37	>50	>50	ND	ND
C		93IN905	>50	>50	>50	>50	>50	>50	>50	>50	>50	>50	>50	>50	>50	>50	>50	>37	>50	>50	>50	>50
		IAVI-C-18	>50	ND	>50	>50	>50	>50	>50	ND	>50	>50	>50	>50	ND	>50	>50	>37	>50	>50	ND	ND
		IAVI-C-22	>50	>50	>50	>50	>50	>50	>50	>50	>50	>50	>50	>50	>50	>50	>50	>37	>50	>50	>50	>50
		IAVI-C-3	16.4	ND	48.1	>50	>50	>50	48.11	ND	>50	>50	>50	>50	ND	>50	>50	>37	>50	>50	ND	ND
		92UG005	>50	>50	>50	>50	>50	>50	>50	>50	>50	>50	>50	>50	>50	>50	>50	>37	>50	>50	>50	>50
		92UG024	28.48	>50	>50	>50	44.42	>50	>50	44.87	>50	>50	47.77	>50	>50	>50	>50	>37	>50	>50	>50	>50
		MGRM-D-001	>50	ND	>50	>50	>50	>50	>50	ND	>50	>50	>50	>50	ND	>50	>50	>37	>50	>50	ND	ND
		MGRM-D-018	>50	ND	>50	>50	>50	>50	>50	ND	>50	>50	>50	>50	ND	>50	>50	>37	>50	>50	ND	ND
CRF01_AE		92TH021	>50	>50	>50	>50	>50	>50	>50	>50	>50	>50	>50	>50	>50	>50	>50	>37	>50	>50	>50	>50
		CMU02	>50	ND	>50	>50	>50	>50	>50	ND	>50	>50	>50	>50	ND	>50	>50	>37	>50	>50	ND	ND
CRF_AG		MGRM-AG-005	>50	>50	>50	>50	>50	>50	>50	>50	>50	>50	>50	>50	>50	>50	>50	>37	>50	>50	>50	>50
Control		aMLV	>50	>50	>50	>50	>50	>50	>50	>50	>50	>50	>50	>50	>50	>50	>50	>37	>50	>50	>50	>50

<1 | 1-5 | 5-10 | 10-25 | >25 μg/ml
