# Supplementary material for: Memory B Cell Antibodies to HIV-1 gp140 Cloned from Individuals Infected with Clade A and B Viruses
Source: PLoS One. 2011 Sep 8;6(9):e24078. doi: 10.1371/journal.pone.0024078 (PMC3169578; doi:10.1371/journal.pone.0024078)
Supplement: Table S4 — Sequence of YU-2 gp120 overlapping peptides. (PDF) [file pone.0024078.s007.pdf]

#	Amino acid sequence	#	Amino acid sequence
1	SAAEQLWVTVYYGVPVWKEA	48	LAEEEIVIRSENFTNNAKTI
2	LWVTVYYGVPVWKEATTTLF	49	IVIRSENFTNNAKTIIVQLN
3	YYGVPVWKEATTTLFCASDA	50	ENFTNNAKTIIVQLNESVVI
4	VWKEATTTLFCASDAKAYDT	51	NAKTIIVQLNESVVINCTRP
5	TTTLFCASDAKAYDTEVHNV	52	IVQLNESVVINCTRPNNNTR
6	CASDAKAYDTEVHNVWATHA	53	ESVVINCTRPNNNTRKSINI
7	KAYDTEVHNVWATHACVPTD	54	NCTRPNNNTRKSINIGPGRA
8	EVHNVWATHACVPTDPNPQE	55	NNNTRKSINIGPGRALYTT
9	WATHACVPTDPNPQEVKLEN	56	RKSINIGPGRALYTTGEII
10	CVPTDPNPQEVKLENVTENF	57	NIGPGRALYTTGEIIGDIRQ
11	PNPQEVKLENVTENFNMWKN	58	RALYTTGEIIGDIRQAHCNL
12	VKLENVTENFNMWKNNMVEQ	59	TGEIIGDIRQAHCNLSKTQW
13	VTENFNMWKNNMVEQMHEDI	60	GDIRQAHCNLSKTQWENTLE
14	NMWKNNMVEQMHEDIISLWD	61	AHCNLSKTQWENTLEQIAIK
15	NMVEQMHEDIISLWDQSLKP	62	SKTQWENTLEQIAIKLKEQF
16	MHEDIISLWDQSLKPCVKLT	63	ENTLEQIAIKLKEQFGNNKT
17	ISLWDQSLKPCVKLTPLCVT	64	EQIAIKLKEQFGNNKTIIFN
18	DQSLKPCVKLTPLCVTLNCT	65	KLKEQFGNNKTIIFNPSS
19	PCVKLTPLCVTLNCTDLRNA	66	EQFGNNKTIIFNPSSGGDPE
20	TPLCVTLNCTDLRNATNTTS	67	NKTIIFNPSSGGDPEIVTHS
21	TLNCTDLRNATNTTSSSWET	68	FNPSSGGDPEIVTHSFNC
22	DLRNATNTTSSSWETMEKGE	69	SSGGDPEIVTHSFNCGGEFF
23	TNTTSSSWETMEKGEIKNCS	70	PEIVTHSFNCGGEFFYCNST
24	SSWETMEKGEIKNCSFNITT	71	HSFNCGGEFFYCNSTQLFTW
25	MEKGEIKNCSFNITTSIRDK	72	GGEFFYCNSTQLFTWNDTRK
26	IKNCSFNITTSIRDKVQKEY	73	YCNSTQLFTWNDTRKLNNT
27	FNITTSIRDKVQKEYALFYN	74	TQLFTWNDTRKLNNTGRNIT
28	SIRDKVQKEYALFYNLDVVP	75	WNDTRKLNNTGRNITLPCRI
29	VQKEYALFYNLDVVPIDNAS	76	KLNNTGRNITLPCRIKQIIN
30	ALFYNLDVVPIDNASYRLIS	77	GRNITLPCRIKQIINMWQEV
31	LDVVPIDNASYRLISCNTSV	78	LPCRIKQIINMWQEVGKAMY
32	IDNASYRLISCNTSVITQAC	79	KQIINMWQEVGKAMYAPPIR
33	YRLISCNTSVITQACPKVSF	80	MWQEVGKAMYAPPIRGQIRC
34	CNTSVITQACPKVSFEPIPI	81	GKAMYAPPIRGQIRCSSNIT
35	ITQACPKVSFEPIPIHYCAP	82	APPIRGQIRCSSNITGLLLT
36	PKVSFEPIPIHYCAPAGFAI	83	GQIRCSSNITGLLLTRDGGK
37	EPIPIHYCAPAGFAILKCND	84	SSNITGLLLTRDGGKDTNGT
38	HYCAPAGFAILKCNDKKFN	85	GLLLTRDGGKDTNGTEIFRP
39	PAGFAILKCNDKKFNGTGPC	86	RDGGKDTNGTEIFRPGGGDM
40	ILKCNDKKFNGTGPCTNVST	87	DTNGTEIFRPGGGDMRDNWR
41	DKKFNGTGPCTNVSTVQCTH	88	EIFRPGGGDMRDNWRSELYK
42	GTGPCTNVSTVQCTHGIRPV	89	GGGDMRDNWRSELYKYKVVK
43	TNVSTVQCTHGIRPVVSTQL	90	RDNWRSELYKYKVVKIEPL
44	VQCTHGIRPVVSTQLLLNGS	91	RSELYKYKVVKIEPLGVAPT
45	GIRPVVSTQLLLNGSLAEEE	92	KYKVVKIEPLGVAPTKAKRR
46	VSTQLLLNGSLAEEEIVIRS	93	KIEPLGVAPTKAKRRVVQRE
47	LLNGSLAEEEIVIRSENFTN	94	GVAPTKAKRRVVQREKR
